# Validation of an optimized in-house enzyme-linked immunosorbent assay for enhanced detection of *Trichinella* spp. exposure in swine

**DOI:** 10.1016/j.fawpar.2026.e00322

**Published:** 2026-02-19

**Authors:** Vladislav A. Lobanov, Kelly A. Konecsni, W. Brad Scandrett

**Affiliations:** Center for Food-borne and Animal Parasitology, Canadian Food Inspection Agency, Saskatoon, Saskatchewan S7N 2R3, Canada

**Keywords:** *Trichinella* infection, ELISA, Western blotting, Serosurveillance, Epidemiology, Commercial swine

## Abstract

The International Commission on Trichinellosis (ICT) and the World Organization for Animal Health (WOAH) recommend the use of an indirect enzyme-linked immunosorbent assay (ELISA) that utilizes excretory-secretory (E-S) antigens (ESA) of *Trichinella spiralis* for surveillance and epidemiological studies in pigs and wild boars. Our efforts to optimize and standardize ESA production and ELISA protocols resulted in improved diagnostic performance of an in-house E*-*S ELISA. To validate the optimized assay, we compared its performance with that of a commercial E-S ELISA kit using sera from a representative set of commercial Canadian pigs (presumably *Trichinella*-free) and pigs experimentally infected with *Trichinella* spp. Both assays correctly identified the positive and negative sera, yielding 100% diagnostic sensitivity and specificity. However, in-house E-S ELISA exhibited a higher resolving power, as evidenced by the markedly better separation of normalized absorbance values of positive sera from those of samples from the negative pig population. Furthermore, significantly higher serial dilutions of sera from pigs experimentally infected with *T. spiralis*, *T. pseudospiralis*, *T. britovi* and *T. nativa* tested positive by the in-house E-S ELISA, demonstrating a higher analytical sensitivity of this assay. We continued testing sera from Canadian commercial pigs using the in-house assay to obtain a more accurate estimate of its diagnostic specificity. A total of 6345 animals have been tested, with only 11 samples showing test values above the cut-off. Ten of these sera tested negative by confirmatory western blot (WB). Therefore, the diagnostic specificity of in-house E-S ELISA alone and in combination with WB testing was 99.84% and 99.98%, respectively. WB detected seroconversion earlier than the optimized E-S ELISA in five of 15 pigs experimentally infected with various low doses of *T. spiralis*. The results of this study support the use of the optimized E-S ELISA and confirmatory WB for epidemiological surveys to monitor exposure to *Trichinella* spp. in swine.

## 1 Introduction

Trichinellosis is a potentially life-threatening zoonotic disease of humans caused by nematodes of the genus *Trichinella*. The genus currently comprises 13 genotypes, 10 of which have been assigned species names (Zarlenga et al., 2020). *Trichinella* spp. complete their direct life cycle in a single host, establishing the infective larval stage in striated muscles. Transmission occurs via the ingestion of muscle tissues containing viable first-stage larvae (L1) (Gottstein et al., 2009; Pozio and Zarlenga, 2013). Natural infections with these parasitic nematodes have been confirmed in over 150 mammalian species across 12 orders (Pozio, 2005). While the distribution of *Trichinella spiralis* and *Trichinella pseudospiralis* can be defined as cosmopolitan, other taxa are restricted to specific geographic regions (Malone et al., 2024; Pozio, 2007).

Historically, most reported cases of trichinellosis have been attributed to the consumption of pork from infected pigs (Murrell and Pozio, 2011). The highest reproductive capacity and larval survival rates of *T. spiralis* in swine make it the only known species capable of persisting in the domestic cycle. Current evidence strongly suggests that *T. spiralis* infections in wildlife hosts (sylvatic cycle) cannot sustain themselves without spillover from the domestic cycle (Hill et al., 2010; Oksanen et al., 2018).

The implementation of post-slaughter testing and strict biosecurity management practices for modern pork production in industrialized countries has reduced the risk of *Trichinella* spp. in commercial swine to a negligible level (Gamble et al., 2019). Apart from *T. pseudospiralis*, very low reproductive capacity was observed in experimentally infected pigs for other sylvatic *Trichinella* spp. found in North America (Kapel, 2000; Kapel and Gamble, 2000). However, a new species, *Trichinella chanchalensis*, has been detected in several carnivorous wildlife hosts in northwestern Canada (Lobanov et al., 2023; Malone et al., 2025; Sharma et al., 2020), for which infectivity for pigs and other relevant livestock species remains to be determined. Despite these advancements, food safety concerns regarding *Trichinella* infection remain a significant regulatory issue impacting the international trade of pork (Gajadhar et al., 2009).

The WOAH and ICT recommend using only direct methods, such as artificial digestion, to detect *Trichinella* L1 in individual carcasses during slaughter inspection, while serological methods are advised for epidemiological monitoring of *Trichinella* exposure in targeted domestic and wildlife populations, primarily pigs and wild boar (Bruschi, 2019; Nockler et al., 2000). Indirect ELISA is the most commonly used method for detecting exposure to *Trichinella* spp. due to its simplicity and suitability for large-scale testing (Gajadhar et al., 2009). The specificity of indirect ELISA for *Trichinella* improved markedly with the use of excretory-secretory antigens (ESA) released into the culture medium by L1 of *T. spiralis* during in vitro incubation (Gamble and Graham, 1984; Gamble et al., 1997). ESA constitute a complex mixture of different proteins whose composition has yet to be exhaustively defined. A fraction of these proteins shares an immunodominant antigen epitope (carbohydrate epitope containing β-tyvelose) that is highly conserved across *Trichinella* taxa, making ESA a universal reagent for detecting exposure to these parasitic nematodes (Robinson and Connolly, 2005). Other published ELISA formats include dot-ELISA (Su and Prestwood, 1991), competitive ELISA utilizing a mouse monoclonal antibody of immunoglobulin (Ig) M (Gamble and Graham, 1984; Gnjatovic et al., 2019) or IgG (Pilfold et al., 2021) class specific to the immunodominant carbohydrate epitope, and a sandwich ELISA for detecting circulating *T. spiralis* antigens in serum (Liu et al., 2013). The ICT recommends an indirect ELISA using ESA as the primary screening test for detecting *Trichinella* exposure in swine and humans, with positive ELISA results to be confirmed by WB. The use of ESA is also recommended when such a confirmatory WB is applied in humans (Bruschi et al., 2019).

In an earlier national survey conducted by the Canadian Food Inspection Agency (CFIA), where sows were tested for exposure to *Trichinella* spp. using a commercial indirect E-S ELISA, we encountered an unexpectedly high number of non-negative test results. An attempt to employ an in-house indirect E-S ELISA did not remedy the problem. Such assay performance prompted further efforts to standardize and optimize our protocols for ESA production, E-S ELISA, and confirmatory WB. These efforts resulted in significantly improved diagnostic performance of these serological assays (Lobanov et al., 2022). This study aimed to validate the optimized in-house E-S ELISA, including a comparison of its diagnostic performance characteristics to those of a validated commercial E-S ELISA (Frey et al., 2009a). Validation data for the in-house WB assay were also generated in this study.

## 2 Materials and methods

### 2.1 E-S antigen

A single ESA preparation produced in 2018 and stored in small aliquots at −70 °C was used in this study. To initiate the culture, L1 were isolated by artificial digestion from Sprague Dawley rats (Charles River), each infected with 5000 L1 of *T. spiralis*. The detailed production protocol was described earlier (Lobanov et al., 2022) and primarily follows the WOAH Terrestrial Manual (WOAH, 2023). Modifications involved concentrating the medium containing released E-S proteins using centrifugal filter units with a 10,000 Da cut-off membrane (instead of the recommended 5000 Da), followed by transferring the proteins into storage buffer [50 mM Tris-HCl (pH 8.0), 150 mM NaCl, 5% (v/v) glycerol, 0.1 mM EDTA, 0.1 mM dithiothreitol, and 5 μl/ml Calbiochem protease inhibitor cocktail set III] using desalting columns (GE Healthcare).

### 2.2 Serum samples

The Canadian commercial swine herd is considered free from *Trichinella* infection (Appleyard et al., 2002; Gajadhar et al., 1997). In this study, we used sera from commercial pigs collected for national swine serosurveys conducted by CFIA. Serum samples were stored at −20 °C upon arrival at the laboratory. In the sample selection process, preference was given to mature breeder pigs because of the increasing likelihood of exposure to *Trichinella* with age.

A set of sera from 55 randomly selected sows sampled in 2011 during the serosurvey, when the suboptimal performance of E-S ELISA was observed, was used to demonstrate the effect of changes to the in-house assay protocol on its performance. The diagnostic specificity of the optimized in-house E-S ELISA was compared to that of the PrioCHECK E-S ELISA (Thermo Fisher Scientific) using sera collected in 2018 from 917 pigs from Quebec (*n* = 496), Ontario (*n* = 99), Manitoba (*n* = 242) and Saskatchewan (*n* = 80). Subsequently, sera collected in 2019–2020 from 5427 commercial pigs from Alberta (*n* = 404), British Columbia (*n* = 356), Manitoba (*n* = 737), Ontario (*n* = 1492), Quebec (*n* = 1810) and Saskatchewan (*n* = 628) were tested by in-house E-S ELISA to obtain a more accurate estimate of the assay's diagnostic specificity.

A panel of positive samples used for comparing the diagnostic sensitivity of in-house E-S ELISA to that of the commercial assay included 88 sera collected from 52 pigs experimentally infected with *T. spiralis* (72 sera from 40 pigs), *T. pseudospiralis* (five sera from three animals), *Trichinella nativa* (five sera from five pigs), *Trichinella britovi* (three sera serially collected from a single animal) or *Trichinella* T6 (three sera from three animals) (Supplementary Table S1). These 88 serum samples included 42 sera from the sample set described below.

To compare the diagnostic performance of in-house E-S ELISA to that of confirmatory WB, sera (*n* = 113) serially collected from 15 weanling pigs from a *Trichinella*-free herd experimentally infected with various doses of *T. spiralis* ranging from 40 to 1000 L1 (Forbes et al., 2004) were tested using both assays.

### 2.3 E-S ELISA

The performance of the optimized in-house E-S ELISA was initially evaluated by testing 55 sera from sows sampled in 2011 using the protocol implemented in earlier studies performed in our laboratory (Appleyard et al., 2002; Forbes et al., 2004) and the optimized protocol (Lobanov et al., 2022). However, ELISA plates coated with ESA, prepared using the optimized procedure (Lobanov et al., 2022), were used for both protocols. To assess the potential contribution of polyreactive IgM to the non-specific reactivity in E-S ELISA performed under the original conditions, the same set of samples was also tested using the optimized protocol, with the secondary antibody replaced with goat anti-pig IgM antibody conjugated to horseradish peroxidase (HRP; AbD Serotec).

In the following experiments, in-house E-S ELISA was performed as described earlier (Lobanov et al., 2022), except that the serum diluent composition was modified by adding skim milk. Nunc MaxiSorp 96-well plates were coated overnight at 2–8 °C with 100 μl/well of 1 μg/ml ESA in 50 mM carbonate/bicarbonate buffer (pH 9.6). The following incubations were performed at room temperature. For blocking, the plates were incubated for 2 h with wells filled with Tris-buffered saline (TBS; 20 mM Tris-HCl (pH 7.4), 150 mM NaCl) containing 2% (w/v) Ig-free bovine serum albumin (BSA; Millipore-Sigma, St. Louis, MO, USA). After three washes with TBS containing 0.05% Tween-20 (Millipore-Sigma; TBST), sera diluted 1:400 in TBST supplemented with 1% (w/v) BSA and 5% (w/v) skim milk (Hardy Diagnostics, Santa Maria, CA, USA) were added in duplicate at 100 μl/well and plates were incubated for 1 h with shaking at 450 rpm. Plates were washed as above and incubated for 1 h (450 rpm) with goat anti-swine IgG (Fc) HRP-conjugated antibody (Bio-Rad) diluted 1:10,000 in TBST/1% BSA. After six washes, plates were incubated for 10 min with 100 μl/well of 3,3',5,5'-Tetramethylbenzidine substrate (SeraCare, Milford, MA, USA), followed by adding 100 μl/well of TMB BlueSTOP (SeraCare). The absorbance was measured at 650 nm using a SpectraMax Plus 384 spectrophotometer and SoftMax Pro 6.2 software (Molecular Devices). The in-house E-S ELISA results were expressed as the sample-to-positive (S/P) ratio, and the cut-off (S/P = 0.292) established in the previous study using samples from over a thousand Canadian sows was applied (Lobanov et al., 2022).

A low-titer positive control serum was introduced in addition to the assay's positive control to assess the repeatability of the in-house E-S ELISA. To prepare this control serum, we assessed serial two-fold dilutions of the *Trichinella*-positive control serum in negative pig serum using the in-house assay and selected a dilution that consistently produced test values marginally above the cut-off. This serum was included on each plate in multiple E-S ELISA runs, serving as a control for between-run repeatability.

PrioCHECK E-S ELISA was performed according to the manufacturer's instructions (Version 1.4_e). Although the kit manual recommended singlicate testing, we tested all samples in duplicate. According to the instructions, test results were expressed as a percentage of positivity (PP) and the provided cut-off value of 15% was applied.

### 2.4 Confirmatory immunoblot

The WB assay was performed as previously described (Lobanov et al., 2022). Each serum sample was incubated with a membrane strip containing two lanes, one with electrophoretically separated protein standards (Precision Plus All-Blue, Bio-Rad) and the other with the ESA preparation. A lane with protein standards was included on each strip to facilitate the determination of molecular masses of reactive bands in the E-S protein complex using Image Lab 6.1.0 software (Bio-Rad). The presence of three closely migrating reactive bands of nearly equal intensity (diagnostic triplet) with molecular masses in the approximate range of 42–55 kilodaltons (kDa) indicated a positive result.

### 2.5 Statistical analysis

Diagnostic specificity (DSp) values with 95% confidence interval (CI) were calculated using the online version of MedCalc (https://www.medcalc.org/; accessed August 31, 2023). The significance of differences in absorbance values obtained from ELISAs performed according to different protocols was assessed using the Mann-Whitney U test in Prism 6.02 (GraphPad). The level of agreement between the in-house E-S ELISA and PrioCHECK E-S ELISA in testing 917 sera from the negative pig population and 88 sera from experimentally infected pigs was categorized using Cohen's kappa, with the aid of the GraphPad QuickCalcs online resource (https://www.graphpad.com/quickcalcs/kappa1/; accessed July 24, 2024). Kappa values were assigned the following strengths of agreement: < 0.00 ‘no agreement’, 0.00–0.20 ‘slight’, 0.21–0.40 ‘fair’, 0.41–0.60 ‘moderate’, 0.61–0.80 ‘substantial’ and 0.81–1.00 ‘almost perfect’ agreement (Landis and Koch, 1977). The degree of correlation between the normalized test values from in-house and PrioCHECK E-S ELISAs was assessed separately for negative and positive samples using Spearman's Rank Correlation. This analysis was performed using the ggplot2 package in R (4.4.1). The strength of the correlation coefficients was interpreted according to the criteria in (Evans, 1996), where coefficient values of 0.00–0.19 are classified as ‘very weak’, 0.20–0.39 ‘weak’, 0.40–0.59 ‘moderate’, 0.60–0.79 ‘strong’, and 0.80–1.00 ‘very strong’.

## 3 Results

**Fig. 1:**
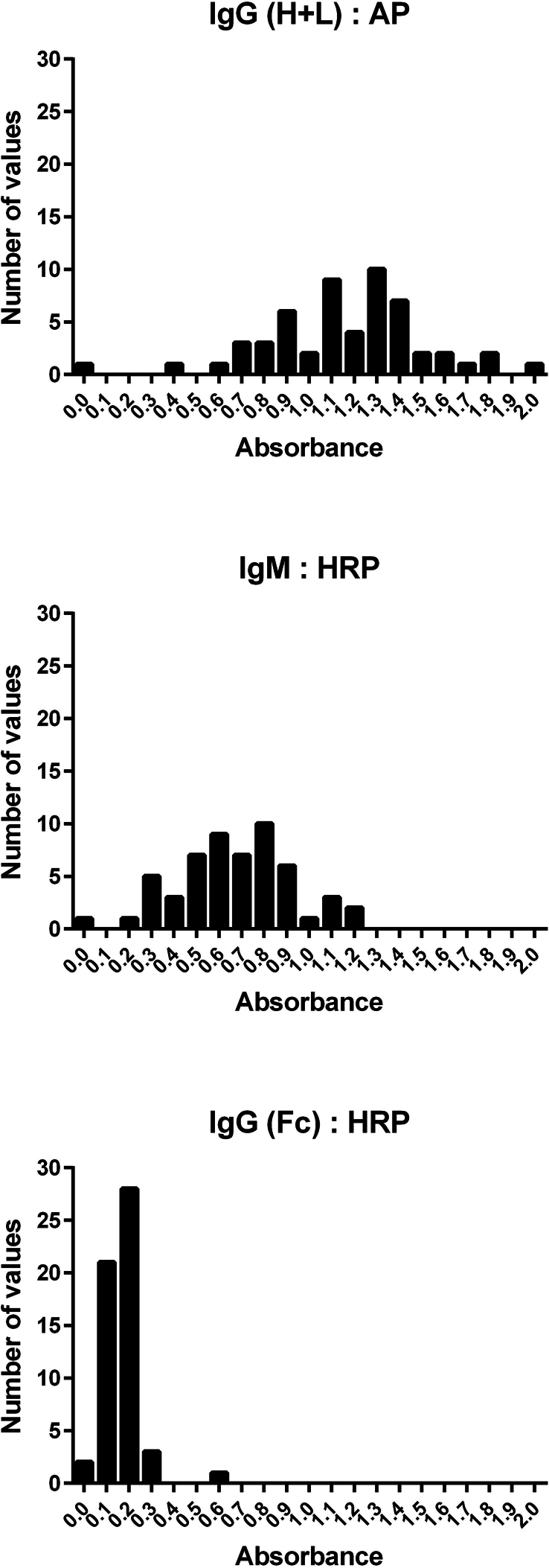
The results of testing 55 sera from presumably *Trichinella*-free commercial pigs sampled in 2011 by E-S ELISA under different conditions. The E-S ELISA was carried out according to the earlier published protocol using a 1:75 serum dilution and anti-pig immunoglobulin (Ig) G (H + L) secondary antibody conjugated to alkaline phosphatase (AP; KPL, Gaithersburg, MD, USA) or according to the optimized protocol with either horse radish peroxidase (HRP)-conjugated secondary antibody specific to porcine IgM or anti-pig IgG (Fc) HRP-conjugated antibody. A strong positive and medium positive control sera from pigs experimentally infected with *Trichinella spiralis*, and a negative control serum were included on each plate. The absorbance values of the positive controls were consistently above 2.0, demonstrating that the improved specificity of the ELISA with anti-pig IgG (Fc) HRP was not due to a significant reduction in sensitivity. The data are presented as a frequency distribution of absorbance values, graphed in GraphPad Prism 6.02. Fc, fragment crystallizable (antibody region). H + L, heavy and light (antibody chains).

Graphs in Fig. 1 illustrate the frequency distribution of absorbance values obtained on testing 55 sera from presumably *Trichinella*-free commercial pigs sampled in 2011 using indirect E-S ELISAs with secondary detection antibodies of varying specificities. A rightward shift towards higher absorbance values was observed in E-S ELISA performed according to the earlier published protocol, which used a secondary antibody specific for the heavy and light (H + L) chains of porcine IgG and a lower test serum dilution. The mean absorbance with a standard deviation (SD) for this data set was 1.159 ± 0.353 (Range: 0.034–1.994). Absorbance values in E-S ELISA with optimized test serum dilution and other conditions, including the use of the secondary conjugated antibody specific to the IgG's fragment crystallizable (Fc) region, were significantly lower (*p* = 0.0001; Mann-Whitney U test); the mean absorbance was only 0.168 ± 0.085 (Range: 0.004–0.643). The absorbance values in E-S ELISA, performed according to the optimized procedure but with an IgM-specific conjugate, were comparatively high, with a mean absorbance of 0.671 ± 0.253 (Range: 0.031–1.193).

**Fig. 2:**
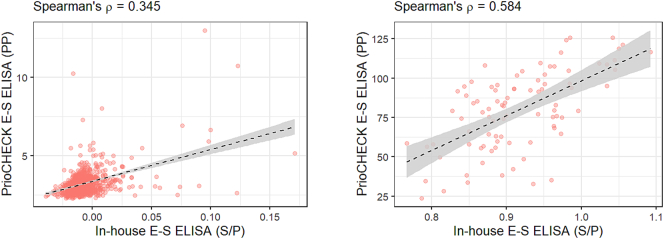
Correlation between normalized test values of in-house E-S ELISA (S/P, sample-to-positive) and PrioCHECK E-S ELISA (PP, percentage of positivity) for negative (*n* = 917; left panel) and positive (*n* = 88; right panel) pig sera. The graphs were created using the ggplot2 package in R (4.4.1). The dashed line represents the linear regression trend, and the shaded gray area indicates the 95% confidence interval. Spearman's rank correlation coefficient values are shown above the panels.

**Fig. 3:**
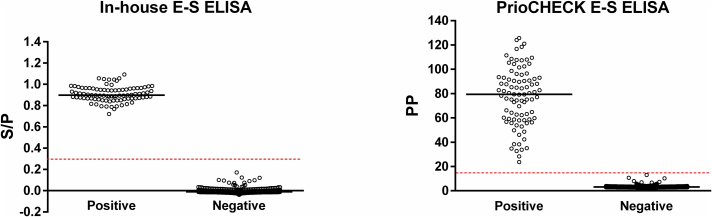
Comparative analysis of the diagnostic performance of in-house and PrioCHECK E-S ELISAs using negative (*n* = 917) and positive (*n* = 88) pig sera. Individual S/P (sample-to-positive) or PP (percentage of positivity) values are depicted as a scatter plot with the mean represented by a solid line (GraphPad Prism 6.02). A red dashed line represents the cut-off for each assay. (For interpretation of the references to colour in this figure legend, the reader is referred to the web version of this article.)

Sera from 917 commercial pigs sampled in 2018 tested negative by the optimized in-house E-S ELISA and PrioCHECK E-S ELISA, resulting in 100% DSp (95% CI: 99.60–100.00) for both assays. The diagnostic sensitivity (DSe) of these assays was also 100% (95% CI: 95.89–100.00), as all 88 sera from experimentally infected pigs tested positive by both assays. The level of agreement between the in-house and commercial E-S ELISAs was categorized using Cohen's kappa statistic as ‘almost perfect’ (K = 1.00; 95% CI: 1.00-1.00). The correlation between normalized test values of the two assays for the negative pig population (*n* = 917) was classified as ‘weak’ (Spearman's *ρ* = 0.345), whereas a ‘moderate’ correlation (Spearman's *ρ* = 0.584) was observed for the positive samples (*n* = 88; Fig. 2). Notably, the in-house E-S ELISA showed greater discriminatory power, with the normalized test values for the positive and negative samples being markedly better separated than in the commercial assay (Fig. 3).

**Fig. 4:**
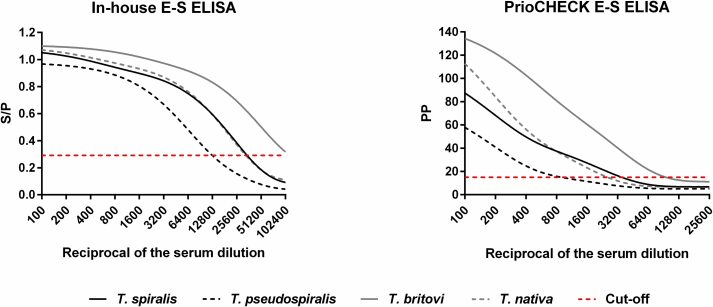
Comparative analysis of analytical sensitivity of in-house and PrioCHECK E-S ELISAs. Serial two-fold dilutions made using 56 days post-infection (DPI) sera from pigs experimentally infected with 30,400 first-stage larvae (L1) of *T. spiralis*, 60,000 L1 of *T. pseudospiralis* or 60,000 L1 of *T. britovi*, and 79 DPI serum from a pig infected with 10,000 L1 of *T. nativa* were tested by both assays. These pigs had 330, 75, 1.65 and 0 larvae per gram in the diaphragm muscle, respectively, as revealed by artificial digestion. Normalized ELISA test values were plotted against serum dilutions starting at a reciprocal of 100 (GraphPad Prism 6.02). The curves were fitted to the data by non-linear regression (two-phase exponential decay). A red dashed line represents the cut-off for each assay. (For interpretation of the references to colour in this figure legend, the reader is referred to the web version of this article.)

The analytical sensitivity of the optimized in-house E-S ELISA was also higher than that of the PrioCHECK assay, as determined by testing serial dilutions of sera collected at or over eight weeks post-inoculation from pigs experimentally infected with high doses of *T. spiralis*, *T. pseudospiralis*, *T. britovi* or *T. nativa* (Fig. 4). All dilutions of the *T. britovi*-infected pig serum up to a reciprocal of 102,400 produced test values above the cut-off in the in-house E-S ELISA. In contrast, in the commercial assay, dilutions of this serum above 1:6400 were negative. Similarly, serum dilutions up to 1:25,600 from pigs infected with *T. spiralis* or *T. nativa* were positive in the in-house assay, whereas in the commercial E-S ELISA, these sera were positive only in dilutions up to 1:1600 for *T. nativa* and 1:3200 for *T. spiralis*. The *T. pseudospiralis*-infected serum was positive in dilutions up to 1:6400 in the in-house assay. However, in the commercial E-S ELISA, dilutions of this serum up to 1:800 produced test values above the cut-off.

**Supplementary Fig. S1:**
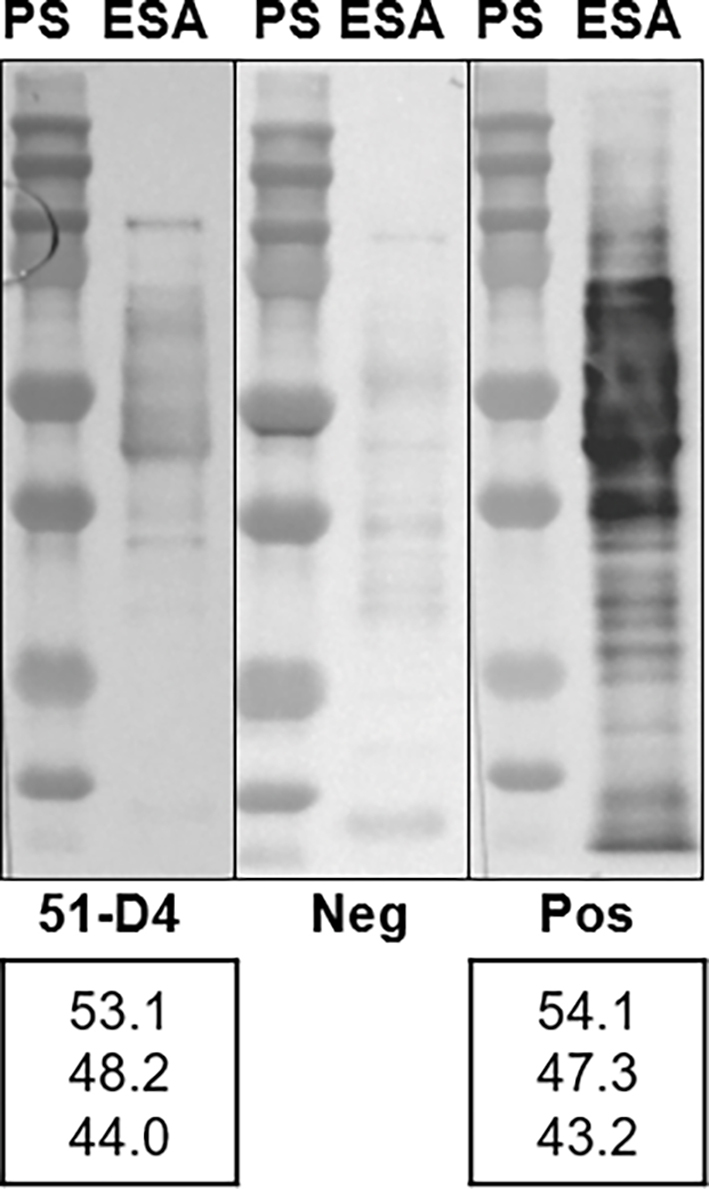
**The binding profile of the E-S ELISA non-negative sample (51-D4) that reacted with the diagnostic triplet bands in the confirmatory western blot.** Pos, membrane strip incubated with positive control serum. PS, protein standards (Precision Plus All-Blue, Bio-Rad). ESA, excretory-secretory antigens. Values at the bottom are the molecular masses (in kilodaltons) of diagnostic triplet bands, determined using Image Lab 6.1.0 software (Bio-Rad).

Continued testing of Canadian commercial pigs sampled in 2019–2020 using the in-house E-S ELISA generated test values above the cut-off for only 11 samples. Combining the in-house E-S ELISA results for both 2018 and 2019–2020 negative pig population sample sets, the 11 of 6344 (0.17%) animals with test values above the assay's cut-off resulted in a DSp of 99.83% (95% CI: 99.69–99.91). Of the 11 positive samples, 10 tested negative using confirmatory WB. A summary of the test results for these 11 samples is in Supplementary Table S2. The immunoblot antibody-binding profile of the sample positive in both in-house E-S ELISA and WB is presented in Supplementary Fig. S1. Thus, the DSp of combined in-house E-S ELISA and confirmatory WB was 99.98% (95% CI: 99.91–100.00).

**Fig. 5:**
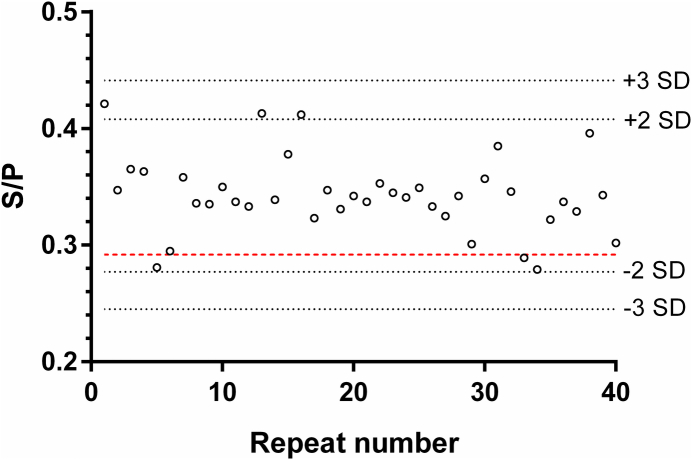
Assessment of the in-house E-S ELISA between-run repeatability. Normalized test values of the low-titer positive control serum from 40 plates were plotted using GraphPad Prism 6.02. Black dotted lines represent ± 2 standard deviations (SD) or ± 3 SD of the mean of this data set. The red dotted line represents the assay's cut-off. (For interpretation of the references to colour in this figure legend, the reader is referred to the web version of this article.)

For the low-titer repeatability control included on 40 ELISA plates, 36 of 40 (90%) normalized test values were within the range represented by two SDs of the mean for this data set; all these values were within the ±3 SD range (Fig. 5). However, three of the 40 values were below the assay's cut-off.

When sera serially collected from 15 weanling pigs experimentally infected with various doses of *T. spiralis* were tested using in-house E-S ELISA and WB, the latter assay enabled earlier detection of seroconversion in five of these animals (Table 1). Data from testing the same samples using E-S ELISA with a ROC-optimized cut-off, as reported in an earlier study conducted in our laboratory, are included in this table. Compared with that study, the optimized E-S ELISA showed improved detection of *T. spiralis* exposure in 12 of these pigs.

**Table 1:** Comparative analysis of the kinetics of antibody responses determined by E-S ELISA or WB in 15 pigs experimentally infected with various doses of *T. spiralis*, ranging from low to moderate.

Animal ID	Oral dose (number of larvae)	LPG #	Seroconversion (DPI)		
			E-S ELISA (this study)	WB	E-S ELISA (Forbes et al., 2004) &
G3P8 (#14)	40	4.8	35	28	42
G3P1 (#11)	40	3.3	42	42	ND
G1P4 (#3)	50	0.005	35	28	35
G1P2 (#1)	50	0.1	35	35	42
G3P7 (#13)	80	2.6	49	42	ND
G3P2 (#12)	120	12.5	42	42	42
G3P10 (#15)	120	3.2	35	35	42
G1P3 (#2)	150	1.1	35	35	56
G1P5 (#4)	150	5.5	35	35	35
G2P46 (#6)	200	43	36	36	44
G1P6 (#5)	300	16.2	35	28	42
G2P47 (#7)	300	72	28	28	35
G2P48 (#8)	400	62	44	35	71
G2P49 (#9)	500	110	28	28	56
G2P50 (#10)	1000	204	28	28	44

LPG, larvae per gramm.

DPI, days post-infection.

ND, not detected.

# Infection levels are presented as mean LPG in the tongue and diaphragm muscle tissue digested separately; these values are from an earlier study (Forbes et al., 2004).

& Results of testing these samples by in-house E-S ELISA with ROC-optimized cut-off in an earlier study performed in our laboratory.

**Supplementary Fig. S2:**
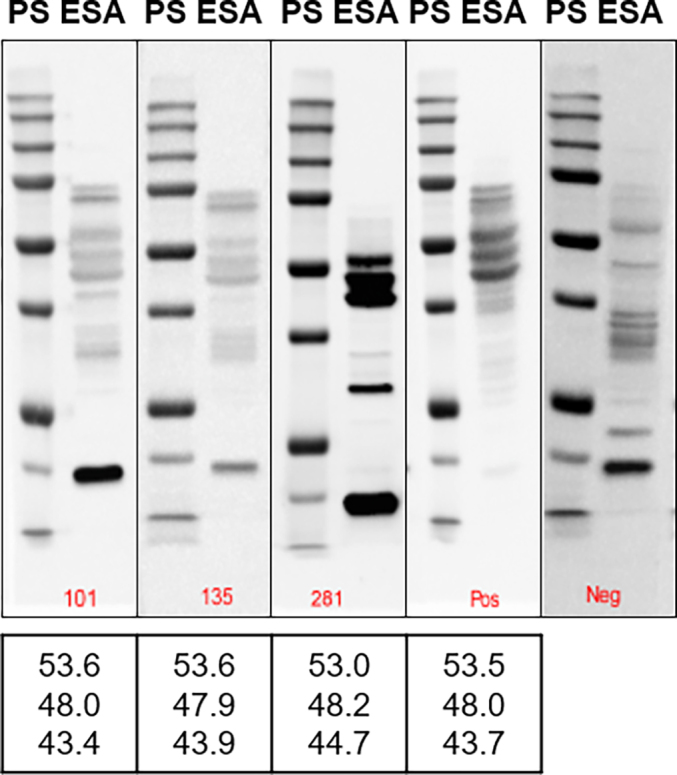
**The binding profiles of sera (101, 135 and 281) that reacted with the diagnostic triplet bands in the assessment of diagnostic specificity of the confirmatory western blot.** Pos, a representative image of the membrane strip incubated with the positive control serum. Neg, a representative image of the membrane strip incubated with the negative control serum. PS, protein standards (Precision Plus All-Blue, Bio-Rad). ESA, excretory-secretory antigens. The bottom values are the molecular masses (in kilodaltons) of the diagnostic triplet bands determined using Image Lab 6.1.0 software (Bio-Rad).

The diagnostic specificity of confirmatory WB was assessed by testing 307 sera selected from the representative set of samples that tested negative in the optimized in-house E-S ELISA in this study. Sera with higher S/P values were preferentially chosen for this analysis. With rare exceptions, these sera exhibited binding to various ESA constituents. However, only 3 of 307 (0.98%) sera produced a characteristic pattern of the dominant triplet of closely migrating bands corresponding to that of the positive control; two closely matched the binding profile of the positive control serum but with comparatively low band intensities, and the third one showed strong binding with the triplet pattern of diagnostic significance but did not display many other minor bands usually present in the positive control (Supplementary Fig. S2).

## 4 Discussion

In a recent study, we generated preliminary performance evaluation data for the optimized in-house E-S ELISA using sera from over a thousand Canadian commercial pigs, a *Trichinella*-free pig population (Lobanov et al., 2022). The assay demonstrated 98% DSp, while 99.9% DSp was achieved after all except one of the non-negative E-S ELISA results were ruled out using confirmatory WB. Additionally, the E-S ELISA correctly identified positive sera from 34 pigs experimentally infected with varying numbers of *T. spiralis* L1. A more comprehensive validation of the in-house E-S ELISA and confirmatory WB performed in this study further demonstrated the robust diagnostic performance of these serological assays, with a DSp of 99.83% for E-S ELISA and 99.98% for combined E-S ELISA and confirmatory WB results in testing 6344 Canadian commercial pigs.

The cause of the increased reactivity of sow sera in commercial and in-house E-S ELISA in the national serosurvey remains uncertain. That same in-house indirect E-S ELISA protocol was used in the serosurvey of Canadian market sows conducted at CFIA in 1996–1997, with only 115 of 14,408 (0.8%) animals testing non-negative (Appleyard et al., 2002). All except three of the non-negative E-S ELISA results were ruled out in that study using a confirmatory competitive ELISA. The increased reactivity observed in sow sera during the earlier national survey was likely attributed to non-specific binding of Ig classes other than G. Our data suggest that polyreactive natural IgM, which typically increases in titer as pigs mature, is a primary contributor to this background reactivity. Polyreactive IgM has a propensity to bind glycan epitopes (Blandino and Baumgarth, 2019; Vollmers and Brandlein, 2006), such as those found in ESA (Ellis et al., 1997). Although the improved diagnostic performance of the in-house E-S ELISA was a cumulative effect of several modifications to its protocol, we believe the primary contributor was replacing the conjugated secondary antibody with specificity to H + L chains of porcine IgG, which would also bind immunoglobulins of other classes with the conjugate that binds the Fc region, mediating specificity restricted mainly to IgG. This change would significantly reduce the detection of IgM binding in the assay.

Other protocol modifications that contributed to reduced background reactivity in the in-house E-S ELISA included supplementing the serum diluent with skim milk and using a higher test-serum dilution, optimized using checkerboard titration. It has been shown that with the increase in storage time of frozen test-negative pig sera, their absorbance values in E-S ELISA tend to drift upward. The increasing background reactivity of those sera was significantly reduced by adding skim milk to the serum diluent (van der Leek et al., 1992). In a deviation from the WOAH-recommended protocol (WOAH, 2023), we omitted skim milk from the wash buffer to prevent clogging the microfluidics of the automated plate washer. However, it was retained in the serum diluent to ensure adequate blocking of non-specific interactions during the primary incubation step. We also included 1% BSA in the serum diluent because synergy between these compounds in reducing background reactivity was observed compared with formulations containing either blocking reagent alone (data not shown).

Our study also demonstrated that using a higher test serum dilution, which further reduced background reactivity in the optimized E-S ELISA, did not negatively affect the assay's sensitivity. In fact, the analytical sensitivity of our optimized E-S ELISA was higher than that of the commercial E-S ELISA, which protocol prescribes a lower serum dilution (i.e., 1:50). These optimizations gave the in-house E-S ELISA strong discriminatory power, producing much clearer separation between positive sera and samples from the negative pig population than the commercial E-S ELISA. However, it should be noted that the data presented here also demonstrated high accuracy of the PrioCHECK E-S ELISA in determining the status of exposure to *Trichinella* in pigs. This is consistent with an earlier published evaluation study for this commercial kit, in which DSe of 97.1–97.8% and DSp of 99.5–99.8% were inferred using Bayesian modelling techniques (Frey et al., 2009a). The lower Spearman's correlation between the normalized test values of the two E-S ELISAs observed in the negative group likely reflects the stochastic nature of the non-specific background signal below the diagnostic cut-off, whereas the moderate correlation in the positive group reflects a more consistent response to *Trichinella*-specific antibodies. Unlike the commercial assay, the in-house E-S ELISA used a normalization strategy that accounts for non-specific binding by subtracting the negative control absorbance value from the sample and positive control absorbances. This could also contribute to the weak correlation for the negative population.

Assigning a negative pig population status to the sampled Canadian commercial pigs based on the assumption that the national commercial swine herd was *Trichinella*-free, without confirming this status in the animals using a gold-standard diagnostic method, was a limitation of this study. However, a negative result for *Trichinella* L1 detection by artificial digestion, considered the gold standard for food safety testing, does not guarantee that the animal tested was not exposed to this parasite. For instance, due to the low reproductive potential in swine of the most prevalent sylvatic *Trichinella* spp. in North America, exposure of pigs to these taxa can induce antibody levels detectable in serological assays for these parasitic nematodes in the absence of L1 detectable in muscle tissues by artificial digestion (Kapel and Gamble, 2000; Nockler et al., 2005). Furthermore, indirect ELISA can be much more sensitive than artificial digestion (Gamble et al., 1983), potentially leading to discrepancies in test results when larval loads are below the detection threshold of the latter method. Although pigs with a true-positive status, determined by the isolation of *Trichinella* L1 via artificial digestion, were the source of positive sera, the lack of samples from naturally exposed animals and the relatively small number of experimentally infected pigs were limitations to estimating the true DSe of the assays evaluated in this study.

Modern controlled production systems ensure a negligible risk of *Trichinella* introduction into commercial swine populations. However, there is growing consumer demand for pork produced in extensive systems, which are perceived as having a lower environmental impact and improved animal welfare. Such production systems (e.g., pasture-based pig operations) inherently carry a higher risk of exposure for pigs to various biosecurity threats, including wildlife species that can harbour *Trichinella* spp. and other pathogens (Pietrosemoli and Tang, 2020). Our detection of a single seropositive (i.e., positive by E-S ELISA and WB) pig in a presumably *Trichinella*-free population aligns with previous findings where pigs kept in controlled production conditions with outdoor access seroconverted after environmental exposure to sylvatic species of *Trichinella* despite testing negative by the gold-standard artificial digestion method (Pozio et al., 2021). This underscores that serology may provide a more sensitive means of detecting biosecurity breaches or exposure to sylvatic *Trichinella* taxa with low reproductive capacity in swine than larval detection via artificial digestion.

Although WB is considered the gold standard for serological confirmation, the possibility of cross-reactivity with tyvelose-bearing immunodominant epitopes of ESA glycoproteins cannot be entirely ruled out. The diagnostic triplet observed in WB is largely driven by antibodies that recognize this rare sugar moiety. While tyvelose is highly specific to *Trichinella* spp., other microorganisms may carry similar molecules. For example, tyvelose was revealed in some bacteria, such as *Salmonella enterica* serogroup D and *Yersinia pseudotuberculosis* (Koropatkin et al., 2003), found in pigs (Laukkanen et al., 2008). In the absence of larval recovery by artificial digestion, the single seropositive case should be interpreted with caution as either a rare environmental exposure to sylvatic *Trichinella* or a cross-reaction of antibodies specific to tyvelose or other carbohydrate epitopes of ESA induced by exposure to other organisms.

Analysis of the 11 E-S ELISA reactors showed that all positive results had relatively low S/P values, with several only slightly above the cut-off. Specifically, the S/P values of 10 samples ruled out by WB ranged from 0.299 to 0.555. Notably, the single confirmed positive serum exhibited an S/P value of 0.333, which was lower than that in eight of the 10 WB-negative samples. This demonstrates that, in a small proportion of animals tested in this study, the optimized in-house E-S ELISA could not distinguish true seroconversion from non-specific background reactivity. This justifies the requirement for a confirmatory test to maintain the high DSp required for serosurveillance. The positive predictive value of serological assays in a pig population considered free from *Trichinella* would be very low. Once such serosurveillance is implemented, seropositive (i.e., positive in E-S ELISA and WB) results should warrant epidemiological follow-up (Frey et al., 2009b). The *Trichinella* infection status of the pig, which was positive by in-house E-S ELISA and confirmed by WB in our study, remains undetermined, as paired muscle tissues were not available for L1 detection by artificial digestion.

In this study, a single ESA preparation produced according to our standardized procedure was used for indirect ELISA and WB. Although crude worm (whole-L1) extract preparations have been shown to be useful as antigens in confirmatory WB for *Trichinella* (Frey et al., 2009b; Nockler et al., 2009), the ESA's simpler composition makes defining the diagnostic protein band-binding pattern in WB more reliable and straightforward. Results of a comprehensive evaluation of diagnostic binding patterns of human and pig sera to ESA in WB have been reported (Gomez-Morales et al., 2012). Sera from pigs that tested negative for *Trichinella* L1 by artificial digestion of at least 50 g of diaphragm muscle but were positive in indirect ELISA with ESA were classified as false-positive in that study. Although those sera reacted with various ESA constituents in WB, none displayed reactivity with the triplet of protein bands with molecular masses in the 48–72 kDa range. Notably, this triplet of closely migrating bands was recognized by all sera (*n* = 120) from naturally and experimentally infected pigs. Similar results were obtained for sera from human trichinellosis cases, further demonstrating the diagnostic value of the protein band triplet. The absolute diagnostic sensitivity and specificity of the immunoblot, with a positive result defined as reactivity to the diagnostic triplet, were reported in that study.

For WB, we used membrane strips with a lane containing marker proteins and a lane of electrophoretically separated ESA proteins to facilitate the determination of molecular masses of ESA-reactive bands using commercial gel/blot image analysis software. In our study, the molecular mass of the diagnostic triplet's protein bands ranged from approximately 43 to 57 kDa. This range is narrower than that reported in the validation study mentioned above (Gomez-Morales et al., 2012) and is similar to that in another study in which WB with ESA was used as a diagnostic tool to detect exposure to *Trichinella* spp. in wild boars (Cuttell et al., 2014). In another study, a triplet of protein bands with molecular masses of 45, 49, and 60 kDa was observed as the most frequently recognized in pigs experimentally infected with *T. spiralis* by WB using ESA (Gondek et al., 2018). Interlaboratory reproducibility of molecular mass estimates for the diagnostic triplet bands may be reduced due to potential variations in assay protocols (e.g., the choice of protein standards) and ESA quality.

In conclusion, the optimized in-house E-S ELISA and confirmatory WB demonstrated robust diagnostic performance, making them reliable tools for epidemiological surveys of *Trichinella* exposure in swine. Such serological surveillance data can contribute to general risk assessments for the pork industry, especially if the monitoring focuses on higher-risk commercial pig populations (e.g., organic operations).

The following are the supplementary data related to this article.

Supplementary Table S1. Serum samples from experimentally infected pigs. Supplementary Table S1

Supplementary Table S2. In-house E-S ELISA positive test results. Supplementary Table S2

## CRediT authorship contribution statement

**Vladislav A. Lobanov:** Visualization, Validation, Resources, Project administration, Methodology, Investigation, Funding acquisition, Formal analysis, Data curation, Conceptualization, Writing – original draft. **Kelly A. Konecsni:** Validation, Investigation, Formal analysis, Data curation, Writing – review & editing. **W. Brad Scandrett:** Validation, Supervision, Resources, Writing – review & editing.

## Declaration of competing interest

The authors declare that they have no known competing financial interests or personal relationships that could have appeared to influence the work reported in this paper.
