# Supplementary material for: Validation of an optimized in-house enzyme-linked immunosorbent assay for enhanced detection of Trichinella spp. exposure in swine
Source: Food Waterborne Parasitol. 2026 Feb 19;42:e00322. doi: 10.1016/j.fawpar.2026.e00322 (PMC13080476; doi:10.1016/j.fawpar.2026.e00322)
Supplement: Supplementary Table S1. Serum samples from experimentally infected pigs. [file mmc3.pdf]

Supplementary Table S1. Serum samples from experimentally infected pigs

№	CFAP ID	Source ID	Species/genotype	Oral dose (L1)	Serum collected (DPI)	LPG	Source
1	R15-72 #427	#427	*T. spiralis*	10,000	149	NA	USDA
2	R15-72 #432	#432	*T. spiralis*	10,000	149	NA	USDA
3	R15-72 #439	#439	*T. spiralis*	10,000	149	NA	USDA
4	R16-47-01	3.8192	*T. spiralis*	30,400	42	330.00	University of Bern
5	R16-47-01	3.8192	*T. spiralis*	30,400	49	330.00	University of Bern
6	R16-47-01	3.8192	*T. spiralis*	30,400	56	330.00	University of Bern
7	R15-55 #1	Pig #1	*T. spiralis*	10,000	79	NA	USDA
8	R15-55 #2	Pig #2	*T. spiralis*	10,000	79	NA	USDA
9	R15-41 Grp1 #6008	Pig #6008	*T. spiralis*	10,000	79	NA	USDA
10	R15-41 #01	Pig #1	*T. spiralis*	10,000	60	NA	USDA
11	R15-41 #02	Pig #2	*T. spiralis*	10,000	60	NA	USDA
12	R15-41 #03	Pig #3	*T. spiralis*	10,000	60	NA	USDA
13	R15-41 #04	Pig #4	*T. spiralis*	10,000	60	NA	USDA
14	R15-41 #05	Pig #5	*T. spiralis*	10,000	60	NA	USDA
15	R15-41 #06	Pig #6	*T. spiralis*	10,000	60	NA	USDA
16	R15-41 #07	Pig #7	*T. spiralis*	10,000	60	NA	USDA
17	R15-41 #08	Pig #8	*T. spiralis*	10,000	60	NA	USDA
18	R15-41 #09	Pig #9	*T. spiralis*	10,000	60	NA	USDA
19	R15-41 #10	Pig #10	*T. spiralis*	10,000	60	NA	USDA
20	R15-41 #11	Pig #11	*T. spiralis*	10,000	60	NA	USDA
21	R06-25 #131	#131	*T. spiralis*	1,000	97	NA	USDA
22	R06-25 #132	#132	*T. spiralis*	1,000	97	NA	USDA
23	R06-25 #133	#133	*T. spiralis*	1,000	97	NA	USDA
24	R06-25 #134	#134	*T. spiralis*	5,000	97	NA	USDA
25	R06-25 #135	#135	*T. spiralis*	5,000	97	NA	USDA
26	R06-25 #138	#138	*T. spiralis*	5,000	97	NA	USDA
27	R06-25 #141	#141	*T. spiralis*	10,000	97	NA	USDA
28	R06-25 #142	#142	*T. spiralis*	10,000	97	NA	USDA
29	R06-25 #143	#143	*T. spiralis*	10,000	97	NA	USDA
30	R06-25 #144	#144	*T. spiralis*	10,000	97	NA	USDA
31	#1 (Forbes et al., 2004)	Grp1-ID2	*T. spiralis*	50	42	0.10	CFIA
32	#1 (Forbes et al., 2004)	Grp1-ID2	*T. spiralis*	50	56	0.10	CFIA
33	#1 (Forbes et al., 2004)	Grp1-ID2	*T. spiralis*	50	63	0.10	CFIA
34	#1 (Forbes et al., 2004)	Grp1-ID2	*T. spiralis*	50	112	0.10	CFIA
35	#2 (Forbes et al., 2004)	Grp1-ID3	*T. spiralis*	150	42	1.10	CFIA
36	#2 (Forbes et al., 2004)	Grp1-ID3	*T. spiralis*	150	56	1.10	CFIA
37	#2 (Forbes et al., 2004)	Grp1-ID3	*T. spiralis*	150	100	1.10	CFIA
38	#2 (Forbes et al., 2004)	Grp1-ID3	*T. spiralis*	150	105	1.10	CFIA
39	#4 (Forbes et al., 2004)	Grp1-ID5	*T. spiralis*	150	42	5.50	CFIA
40	#4 (Forbes et al., 2004)	Grp1-ID5	*T. spiralis*	150	56	5.50	CFIA
41	#4 (Forbes et al., 2004)	Grp1-ID5	*T. spiralis*	150	126	5.50	CFIA
42	#5 (Forbes et al., 2004)	Grp1-ID6	*T. spiralis*	300	42	16.20	CFIA
43	#5 (Forbes et al., 2004)	Grp1-ID6	*T. spiralis*	300	63	16.20	CFIA
44	#5 (Forbes et al., 2004)	Grp1-ID6	*T. spiralis*	300	147	16.20	CFIA
45	#6 (Forbes et al., 2004)	Grp2-ID46	*T. spiralis*	200	44	43.00	CFIA
46	#6 (Forbes et al., 2004)	Grp2-ID46	*T. spiralis*	200	56	43.00	CFIA
47	#6 (Forbes et al., 2004)	Grp2-ID46	*T. spiralis*	200	71	43.00	CFIA
48	#6 (Forbes et al., 2004)	Grp2-ID46	*T. spiralis*	200	146	43.00	CFIA
49	#7 (Forbes et al., 2004)	Grp2-ID47	*T. spiralis*	300	44	72.00	CFIA
50	#7 (Forbes et al., 2004)	Grp2-ID47	*T. spiralis*	300	56	72.00	CFIA
51	#7 (Forbes et al., 2004)	Grp2-ID47	*T. spiralis*	300	71	72.00	CFIA
52	#7 (Forbes et al., 2004)	Grp2-ID47	*T. spiralis*	300	125	72.00	CFIA
53	#8 (Forbes et al., 2004)	Grp2-ID48	*T. spiralis*	400	71	62.00	CFIA
54	#8 (Forbes et al., 2004)	Grp2-ID48	*T. spiralis*	400	118	62.00	CFIA
55	#9 (Forbes et al., 2004)	Grp2-ID49	*T. spiralis*	500	44	110.00	CFIA
56	#9 (Forbes et al., 2004)	Grp2-ID49	*T. spiralis*	500	56	110.00	CFIA
57	#9 (Forbes et al., 2004)	Grp2-ID49	*T. spiralis*	500	71	110.00	CFIA
58	#9 (Forbes et al., 2004)	Grp2-ID49	*T. spiralis*	500	111	110.00	CFIA
59	#10 (Forbes et al., 2004)	Grp2-ID50	*T. spiralis*	1,000	44	204.00	CFIA
60	#10 (Forbes et al., 2004)	Grp2-ID50	*T. spiralis*	1,000	56	204.00	CFIA
61	#10 (Forbes et al., 2004)	Grp2-ID50	*T. spiralis*	1,000	71	204.00	CFIA
62	#10 (Forbes et al., 2004)	Grp2-ID50	*T. spiralis*	1,000	83	204.00	CFIA
63	#12 (Forbes et al., 2004)	Grp3-ID2	*T. spiralis*	120	42	12.50	CFIA
64	#12 (Forbes et al., 2004)	Grp3-ID2	*T. spiralis*	120	49	12.50	CFIA
65	#12 (Forbes et al., 2004)	Grp3-ID2	*T. spiralis*	120	56	12.50	CFIA

66	#12 (Forbes et al., 2004)	Grp3-ID2	*T. spiralis*	120	61	12.50	CFIA
67	#14 (Forbes et al., 2004)	Grp3-ID8	*T. spiralis*	40	56	4.80	CFIA
68	#14 (Forbes et al., 2004)	Grp3-ID8	*T. spiralis*	40	61	4.80	CFIA
69	#15 (Forbes et al., 2004)	Grp3-ID10	*T. spiralis*	120	42	3.20	CFIA
70	#15 (Forbes et al., 2004)	Grp3-ID10	*T. spiralis*	120	49	3.20	CFIA
71	#15 (Forbes et al., 2004)	Grp3-ID10	*T. spiralis*	120	56	3.20	CFIA
72	#15 (Forbes et al., 2004)	Grp3-ID10	*T. spiralis*	120	61	3.20	CFIA
73	R15-41 Pig #490M-OR	Pig #490M-OR	*T. nativa*	10,000	55	0.16	USDA
74	R15-41 Pig #515M-OR	Pig #515M-OR	*T. nativa*	10,000	55	1.28	USDA
75	R15-55 #302	Pig #302	*T. nativa*	10,000	79	NA	USDA
76	R15-41 Grp4 #4061	Pig #4061	*T. nativa*	10,000	72	NA	USDA
77	R15-41 Grp5 #5071	Pig #5071	*T. nativa*	10,000	72	NA	USDA
78	R16-47-03	3.0948	*T. britovi*	60,000	42	1.65	University of Bern
79	R16-47-03	3.0948	*T. britovi*	60,000	49	1.65	University of Bern
80	R16-47-03	3.0948	*T. britovi*	60,000	56	1.65	University of Bern
81	R16-47-02	3.0952	*T. pseudospiralis*	60,000	42	75.00	University of Bern
82	R16-47-02	3.0952	*T. pseudospiralis*	60,000	49	75.00	University of Bern
83	R16-47-02	3.0952	*T. pseudospiralis*	60,000	56	75.00	University of Bern
84	R15-41 Grp4 #5030	Pig #5030	*T. pseudospiralis*	10,000	72	NA	USDA
85	R15-41 Grp4 #5055	Pig #5055	*T. pseudospiralis*	10,000	72	NA	USDA
86	R15-55 #604	Pig #604	*Trichinella* T6	10,000	81	NA	USDA
87	R15-41 Grp2 #5003	Pig #5003	*Trichinella* T6	10,000	72	NA	USDA
88	R15-41 Grp2 #5084	Pig #5084	*Trichinella* T6	10,000	72	NA	USDA

NA, not available
USDA, U.S. Department of Agriculture
L1, first-stage larvae
DPI, days post-infection
LPG, larvae per gramm
CFIA, Canadian Food Inspection Agency
CFAP, Center for Food-borne and Animal Parasitology (CFIA)
