# Supplementary material for: Validation of an optimized in-house enzyme-linked immunosorbent assay for enhanced detection of Trichinella spp. exposure in swine
Source: Food Waterborne Parasitol. 2026 Feb 19;42:e00322. doi: 10.1016/j.fawpar.2026.e00322 (PMC13080476; doi:10.1016/j.fawpar.2026.e00322)
Supplement: Supplementary Table S2. In-house E-S ELISA positive test results. [file mmc4.pdf]

Supplementary Table S2. In-house E-S ELISA positive test results

№	CFAP ID	CSS Sample ID	Other ID	Province	OD (H+)	OD (L+)	OD (NEG)	OD (SAMPLE)	S/P	ELISA Result	WB Result
1	R19-024	AH-2019-CSS-025-202	11-C3	ON	1.716	NA	0.056	0.553	0.299	Positive	Negative
2	R19-036	AH-2019-CSS-046-088	15-G3	SK	1.648	NA	0.067	0.754	0.434	Positive	Negative
3	R19-059	AH-2019-CSS-104-038	33-D3	SK	1.575	NA	0.055	0.822	0.505	Positive	Negative
4	R19-069	AH-2019-CSS-161-002	44-H9	MB	1.837	0.835	0.130	0.836	0.414	Positive	Negative
5	R19-071	AH-2019-CSS-185-029	51-D4	MB	1.682	0.602	0.081	0.615	0.333	Positive	Positive
6	R19-071	AH-2019-CSS-188-011	53-G3	QC	1.743	0.672	0.074	0.998	0.554	Positive	Negative
7	R19-071	AH-2019-CSS-188-014	53-B4	QC	1.743	0.672	0.074	0.592	0.310	Positive	Negative
8	R19-079	AH-2019-CSS-197-006	54-A5	ON	1.727	0.647	0.087	0.678	0.360	Positive	Negative
9	R20-001	AH-2019-CSS-216-038	61-F6	SK	2.042	0.775	0.103	1.179	0.555	Positive	Negative
10	R20-001	AH-2019-CSS-217-060	62-B12	SK	1.938	0.726	0.088	0.917	0.448	Positive	Negative
11	R20-001	AH-2019-CSS-218-036	63-F10	MB	2.075	0.695	0.099	0.773	0.341	Positive	Negative

NA, not available
ON, Ontario
SK, Saskatchewan
MB, Manitoba
QC, Quebec
OD, optical density
H+, high-positive control
L+, low-positive control
S/P, sample-to-positive (normalized test result)
CSS, Canadian Swine Serosurvey
WB, western blot
